# Platelets exacerbate cardiovascular inflammation in a murine model of Kawasaki disease vasculitis

**DOI:** 10.1172/jci.insight.169855

**Published:** 2023-07-24

**Authors:** Begüm Kocatürk, Youngho Lee, Nobuyuki Nosaka, Masanori Abe, Daisy Martinon, Malcolm E. Lane, Debbie Moreira, Shuang Chen, Michael C. Fishbein, Rebecca A. Porritt, Bernardo S. Franklin, Magali Noval Rivas, Moshe Arditi

**Affiliations:** 1 Department of Pediatrics, Division of Infectious Diseases and Immunology, Guerin Children’s at Cedars-Sinai Medical Center, Los Angeles, California, USA.; 2 Infectious and Immunologic Diseases Research Center (IIDRC), Department of Biomedical Sciences, Cedars-Sinai Medical Center, Los Angeles, California, USA.; 3 Department of Pathology, David Geffen School of Medicine at UCLA, Los Angeles, California, USA.; 4 Institute of Innate Immunity, Medical Faculty, University of Bonn, Bonn, Germany.; 5 Smidt Heart Institute, Cedars-Sinai Medical Center, Los Angeles, California, USA.

**Keywords:** Inflammation, Vascular Biology, Cardiovascular disease, Platelets, Vasculitis

## Abstract

Kawasaki disease (KD) is the leading cause of acquired heart disease among children. Increased platelet counts and activation are observed during the course of KD, and elevated platelet counts are associated with higher risks of developing intravenous immunoglobulin resistance and coronary artery aneurysms. However, the role of platelets in KD pathogenesis remains unclear. Here, we analyzed transcriptomics data generated from the whole blood of patients with KD and discovered changes in the expression of platelet-related genes during acute KD. In the *Lactobacillus casei* cell wall extract (LCWE) murine model of KD vasculitis, LCWE injection increased platelet counts and the formation of monocyte-platelet aggregates (MPAs), upregulated the concentration of soluble P-selectin, and increased circulating thrombopoietin and interleukin 6 (IL-6). Furthermore, platelet counts correlated with the severity of cardiovascular inflammation. Genetic depletion of platelets (*Mpl^–/–^* mice) or treatment with an anti-CD42b antibody significantly reduced LCWE-induced cardiovascular lesions. Furthermore, in the mouse model, platelets promoted vascular inflammation via the formation of MPAs, which likely amplified IL-1B production. Altogether, our results indicate that platelet activation exacerbates the development of cardiovascular lesions in a murine model of KD vasculitis. These findings enhance our understanding of KD vasculitis pathogenesis and highlight MPAs, which are known to enhance IL-1B production, as a potential therapeutic target for this disorder.

## Graphical abstract

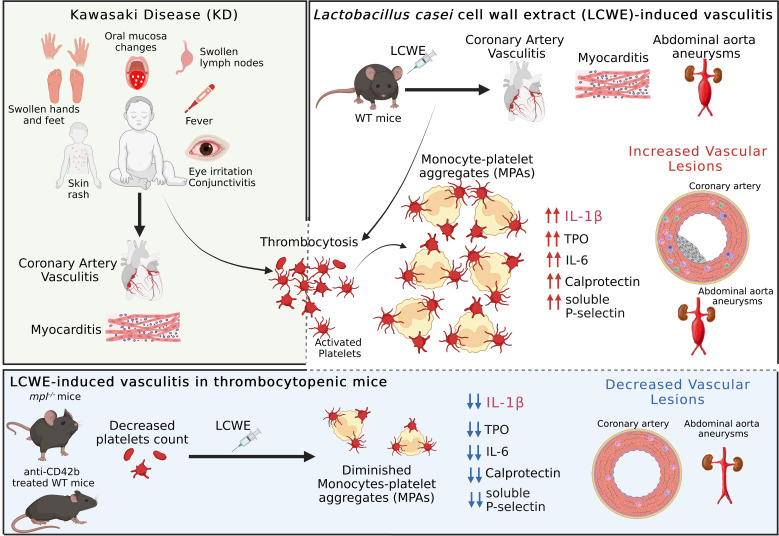

## Introduction

Kawasaki disease (KD), an acute febrile disease and systemic vasculitis, is primarily reported in children younger than 5 years (1, 2). Although the first KD diagnosis was made more than 50 years ago, the KD-triggering agent(s) remains unidentified; however, the disease is suspected of having an infectious origin (3). KD is the leading cause of acquired heart disease in children in developed countries. KD predominantly affects small- and medium-sized vessels, particularly coronary arteries (CAs), and results in the development of CA aneurysms in up to 25% of untreated children (1, 2). CA aneurysms may lead to thrombotic occlusion because of abnormal blood flow conditions in areas of severe dilation (1). Other factors contributing to thrombosis during KD are thrombocytosis, increased platelet activation, adhesion, and endothelial cell dysfunction (1). Furthermore, the ongoing vascular remodeling may lead to coronary stenosis, myocardial ischemia, and even infarction and sudden death (1). The standard of care for patients with KD is a single dose of intravenous immunoglobulin (IVIG) and high-to-moderate dose aspirin in the first 10 days of disease onset. This treatment reduces inflammation and decreases the incidence of CA aneurysms to 3%–5% (1). However, up to 20% of patients with KD are IVIG resistant and at higher risk of developing CA aneurysms, indicating a need for additional therapeutic approaches (1, 2, 4).

Patients with acute KD also exhibit elevated levels of circulating interleukin IB (IL-1B), which are reduced following IVIG treatment (5, 6). Indeed, the analysis of whole blood of patients with KD indicates increased gene expression related to the NLRP3/IL-1B pathway (7). IL-1B is a proinflammatory cytokine crucial for host defense against infections; however, dysregulated IL-1B production is associated with the pathogenesis of several inflammatory disorders (8). Among immune cells, macrophages and monocytes are a major source of IL-1B (8, 9). While IVIG is the standard of care for patients with KD, multiple case reports and a phase II open-label study demonstrated the safety and efficacy of blocking the IL-1 pathway using anakinra, an IL-1 receptor antagonist, to treat KD patients who are refractory to IVIG treatment (10–13).

Platelets and platelet-mediated release of granules, microparticles, and mediators from activated platelets can influence vascular function and may contribute to the remodeling of the vasculature (14–17). Such remodeling may include narrowing of the vessel wall or destruction of proteins in extracellular matrix such as collagen and elastin, and remodeling the vascular intimal endothelial cells (14–17). In addition, growing evidence indicates that platelets, critical mediators of hemostasis and thrombosis (18), also interact with innate immune cells (19), and endothelial cells, to amplify inflammation and impact the pathogenesis of several vascular inflammatory disorders, including atherosclerosis and myocardial infarction (20–23). Platelets can boost NLRP3 inflammasome activation and IL-1B production in human monocytes through a non–cell-to-cell-contact–dependent mechanism via the release of soluble mediators (19). Activated platelets can also aggregate with leukocytes (leukocyte-platelet aggregates), boosting leukocytes’ proinflammatory functions (21, 24, 25). In particular, activated platelets upregulate P-selectin (CD62P) at their surface, which binds with P-selectin glycoprotein ligand 1 (PSGL1) on monocytes, resulting in the formation of monocyte-platelet aggregates (MPAs) (26–28). Indeed, accumulation of MPAs in the circulation has been proposed to represent a robust biomarker of platelet activation (22, 23). By forming MPAs, platelets enhance monocyte inflammatory functions (19, 29), and blood MPAs are increased in cardiovascular and inflammatory diseases (23, 30). Platelets release mediators that boost the expression and secretion of proinflammatory cytokines by human monocytes, such as IL-1B, which are highly inflammatory and important mediators of cardiovascular diseases (31), including KD vasculitis (32).

Increased platelet count, or thrombocytosis, is a common feature of patients with KD and is typically reported in the second to third week after disease onset, usually when CA aneurysms appear (1, 33, 34). In addition, higher peak platelet count has been observed in patients with CA aneurysms and IVIG resistance (35, 36). Patients with KD exhibit enhanced platelet aggregation and increased expression of platelet activation markers, such as platelet-derived microparticles, vascular endothelial growth factor (VEGF), platelet factor 4 (PF4), and β-thromboglobulin (37–43). The frequencies of MPAs and neutrophil-platelet aggregates are increased in patients with KD during the acute phase of the disease (43, 44). However, if and how platelets contribute to the pathogenesis and cardiovascular lesions associated with KD remains unclear.

The *Lactobacillus casei* cell wall extract (LCWE) murine model of KD vasculitis closely mimics the important histopathological, functional, and immune features of human KD vasculitis (32). As observed in patients with KD, the LCWE model is also dependent on the NLRP3/IL-1B axis, and blocking the IL-1 pathway genetically or using anakinra reduces the severity of the disease (7, 45, 46). Previous studies indicate that in LCWE-induced KD vasculitis, monocytes and macrophages are the main sources of IL-1B (46, 47), while neutrophils and eosinophils also express transcripts (*Il1b*, *Nlrp3*, *Casp1*, and *Pycard*) and have the capacity to produce IL-1B (7). Analysis of autopsied heart tissues from patients with KD and cardiovascular lesions of LCWE-injected mice indicates infiltrations of neutrophils, monocytes, and macrophages into the inflamed CA, which can produce IL-1B (7, 46–49). Here, we investigated the role of platelets in LCWE-induced KD vasculitis and found increased platelet counts in the first 2 weeks, which correlated with the severity of the cardiovascular lesions. Mice with LCWE-induced KD vasculitis showed increased frequencies of MPAs associated with elevated levels of circulating IL-1B. Depleting platelets in mice either by using anti-CD42b antibody or using thrombocytopenic *Mpl^–/–^* mice resulted in decreased severity of LCWE-induced KD vasculitis and cardiovascular lesions. Of interest, analysis of human transcriptomics data generated from the whole blood of patients with KD also showed increased expression of genes associated with platelets and their activation. Thus, our studies suggest that platelets contribute to the development of cardiovascular lesions in the LCWE model, potentially by upregulating IL-1B production via MPA formation. This work provides a rationale for antiplatelet therapy and targeting MPAs to inhibit the development and progression of cardiovascular lesions associated with KD vasculitis.

## Results

### Increased expression of platelet-related genes in patients with KD.

**Figure 1:**
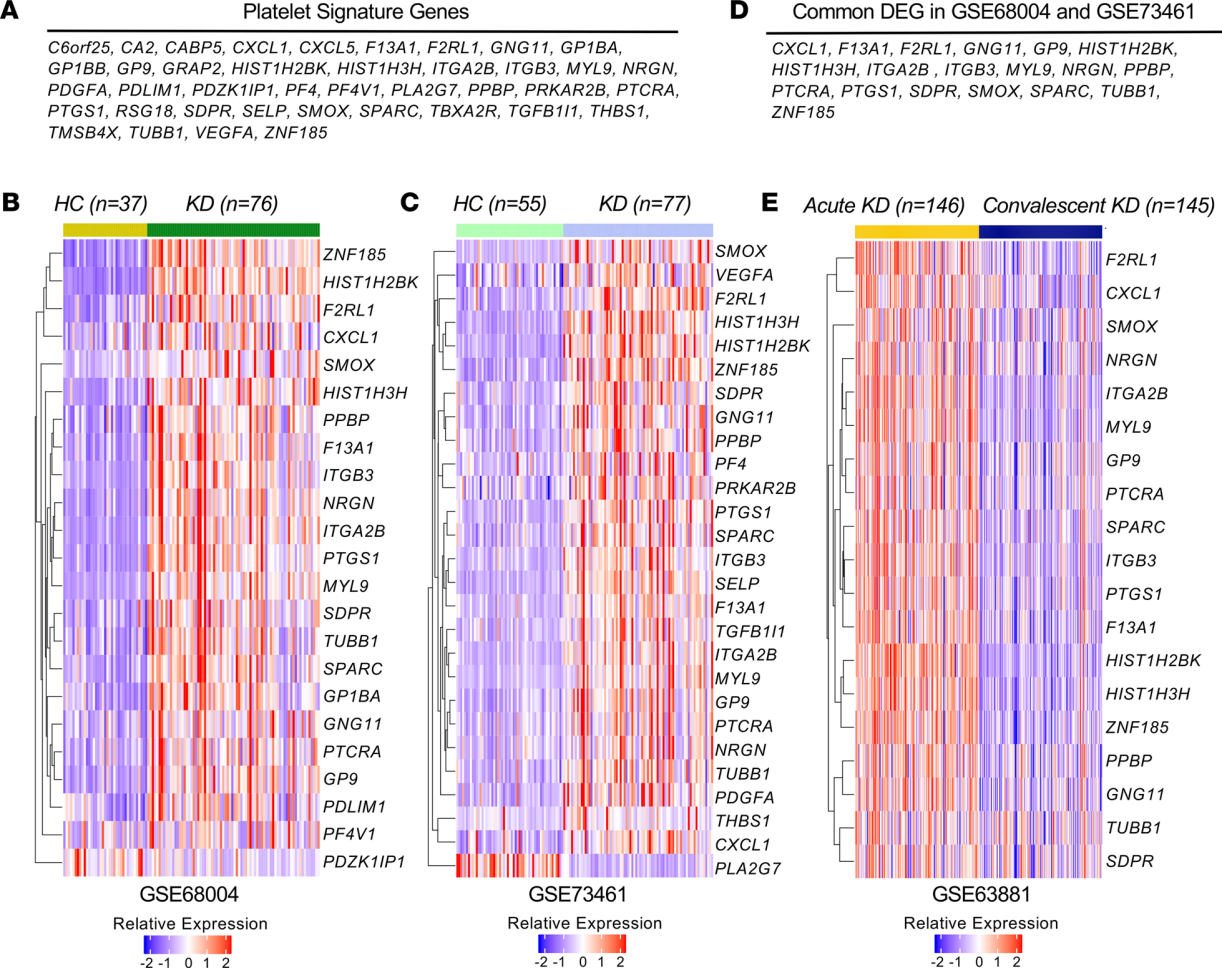
Change in the expression of platelet signature genes during acute KD. (**A**) List of the 40 genes either expressed by platelets or associated with platelet activity included in the platelet signature genes. (**B** and **C**) Heatmaps showing platelet signature genes that are differentially expressed (at least 1.5-fold change [FC] in either direction and with an adjusted *P* value < 0.05) in whole blood from patients with acute KD compared with HCs (**B**: GSE68004, *n* = 76 KD patients and *n* = 37 HCs, and **C**: GSE73461, *n* = 77 KD patients and *n* = 55 HCs). (**D**) List of 19 genes from the platelet signature genes that were commonly upregulated during acute KD in **B** and **C**. (**E**) Expression of common platelet signature differentially expressed genes (DEGs) (**D**) in whole blood of patients with acute KD compared with convalescent IVIG-treated KD patients (GSE63881; *n* = 146 acute KD patients and *n* = 145 convalescent KD patients). Genes were selected based on an adjusted *P* value of less than 0.05. (**B**, **C**, and **E**) Blue–red color gradient: Low to high expression relative to the mean of each row. Each column represents 1 patient of the defined groups. Differential expression was analyzed with GEO2R. HC, healthy control; KD, Kawasaki disease.

To assess the contribution of platelets to KD vasculitis, we used publicly available gene expression data sets generated from patients with KD to analyze the expression of a platelet gene signature comprising 40 genes either expressed by platelets or associated with platelet activity (Figure 1A) (19, 50, 51). Using 2 different transcriptomic data sets generated from whole blood of patients with acute KD and healthy controls (HCs) (NCBI Gene Expression Omnibus [GEO] GSE68004 and GSE73461) (52, 53), we observed that 25 and 29 genes from the platelet gene signature were differentially expressed (adjusted *P* < 0.05). Among these differentially expressed genes, 22 and 26 were upregulated in patients with acute KD (fold change [FC] > 1.5) when compared with controls (Figure 1, B and C). We next identified 19 of the platelet signature genes that were significantly and commonly upregulated during acute KD in these 2 data sets (GSE68004 and GSE73461) (Figure 1D). Using gene expression data generated from whole blood of patients with acute KD and IVIG-treated convalescent KD patients (GSE63881) (54), we observed that these 19 genes commonly upregulated during acute KD were differentially expressed (adjusted *P* < 0.05) in IVIG-treated convalescent patients (Figure 1E). The abundance of platelet-specific and associated transcripts in pediatric KD vasculitis indicates increased platelet activity and warrants further investigation into their role in KD pathogenesis.

### Increased platelet count during LCWE-induced KD vasculitis.

**Figure 2:**
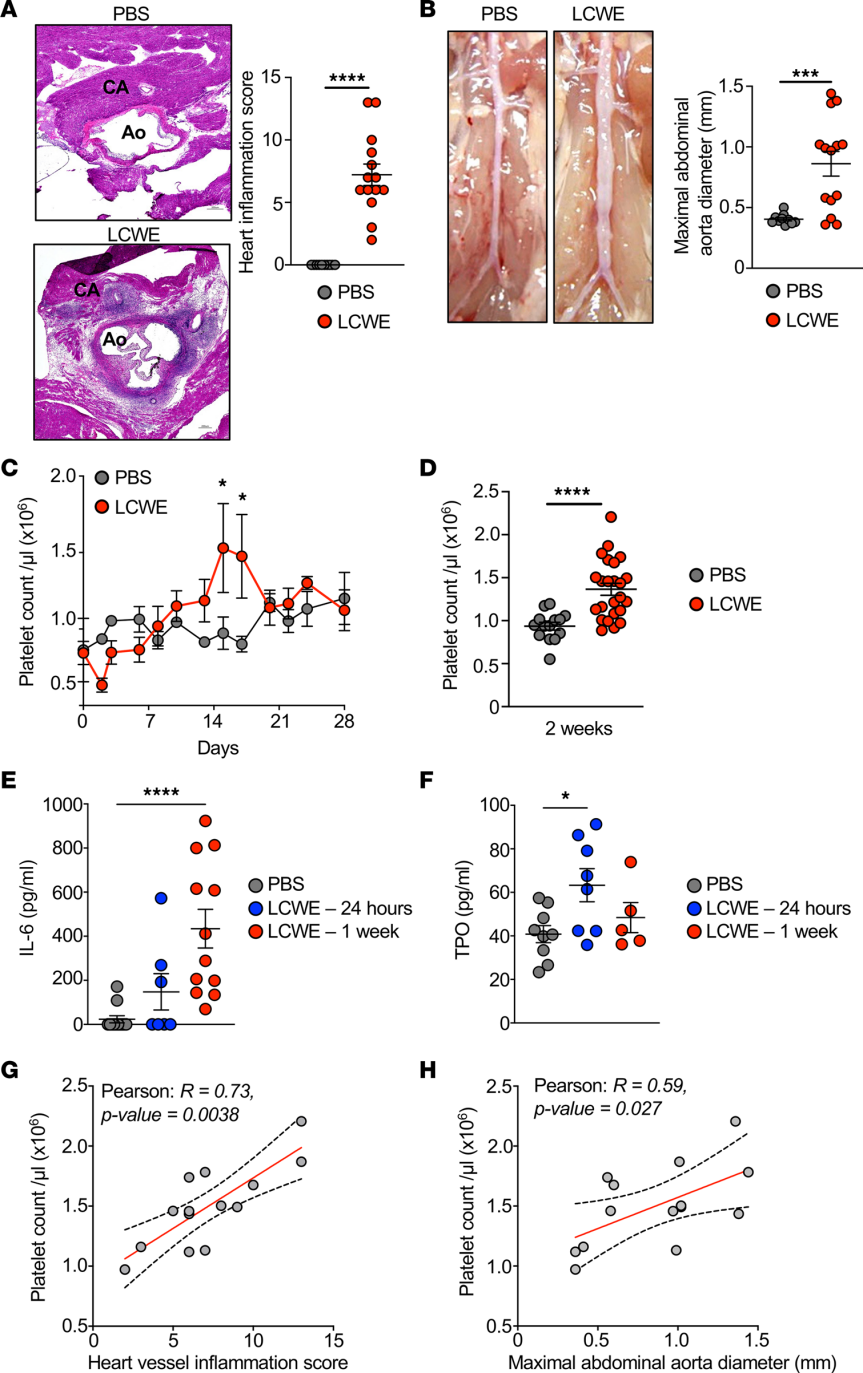
Platelet count correlates with the severity of LCWE-induced KD vasculitis. (**A**) H&E-stained heart tissue sections and heart vessel inflammation score from PBS- and LCWE-injected mice at 2 weeks after LCWE injection (*n* = 10 to 14 mice/group). Scale bar: 200 μm. (**B**) Representative pictures of the abdominal aorta area and maximal abdominal aorta diameter of PBS- and LCWE-injected mice at 2 weeks after LCWE injection (*n* = 10 to 14 mice/group). (**C**) Four-week time-course analysis of platelet counts in the blood of PBS- and LCWE-injected mice (*n* = 4 to 5 mice/group). (**D**) Blood platelet count of PBS- and LCWE-injected mice at 2 weeks after injection (*n* = 14 to 24 mice/group). (**E** and **F**) IL-6 and TPO levels in the serum of PBS- and LCWE-injected mice (*n* = 8 to 12 mice/group) at 24 hours and 1 week after LCWE injection. (**G** and **H**) Correlation of blood platelet counts with heart inflammation score (**G**) and maximal abdominal aorta diameter (**H**). Each symbol represents 1 mouse. Results presented as mean ± SEM. *P < 0.05, ***P < 0.001, ****P < 0.0001 obtained by unpaired 2-tailed Student’s *t* test with Welch’s correction (**A**, **B**, and **D**), 2-way ANOVA with Bonferroni’s multiple-comparison test (**C**), 1-way ANOVA with Tukey’s multiple-comparison test (**E**), Kruskal-Wallis with Dunn’s multiple-comparison test (**F**), or Pearson’ *r* correlation test (**G** and **H**). CA, coronary artery; Ao, Aorta; TPO, thrombopoietin.

To further characterize the contribution of platelets to cardiovascular lesion development during KD, we used the LCWE-induced murine model of KD vasculitis. As previously published (7, 45, 46, 55), compared with PBS-injected control mice, LCWE injection resulted in intense heart vessel inflammation characterized by the development of aortitis and coronary arteritis (Figure 2A). LCWE injection also led to the development of infrarenal abdominal aorta aneurysms and dilations (Figure 2B). A time-course analysis indicated that LCWE-injected mice had increased platelet counts starting 1 week after LCWE injection and peaking on day 14 after LCWE (Figure 2, C and D), similar to previous findings described in KD patients, who show thrombocytosis in the second and third weeks after illness (34). We next measured the circulating levels of IL-6 and thrombopoietin (TPO), both proteins involved in inflammatory thrombocytosis (56, 57). In patients with KD, IL-6 and TPO levels increase during the first week of illness before the platelet count peaks (58). Similarly, compared with PBS-injected control mice, LCWE-injected mice showed significantly increased levels of circulating levels of IL-6 and TPO at 1 week and 1 day after LCWE, respectively (Figure 2, E and F). Finally, in LCWE-injected mice, platelet counts positively and significantly correlated with the development of heart inflammation and abdominal aorta aneurysms (Figure 2, G and H). Taken together, these results indicate that LCWE-induced KD vasculitis and cardiovascular lesion development are associated with elevated platelet counts.

### Platelet depletion decreases the severity of LCWE-induced cardiovascular lesions.

**Figure 3:**
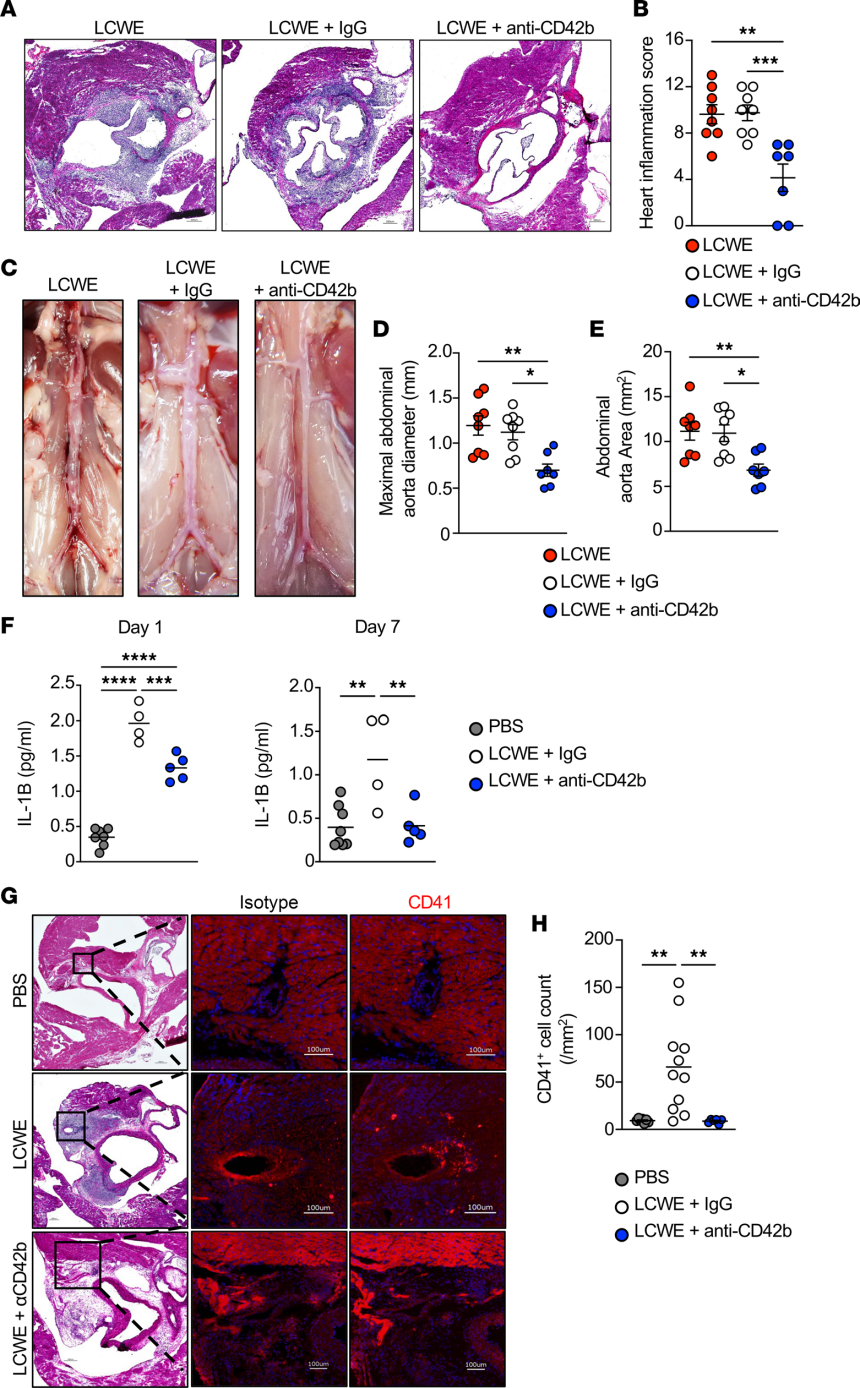
Platelet depletion attenuates the severity of LCWE-induced KD vasculitis. (**A** and **B**) Representative H&E-stained heart tissue sections (**A**) and heart vessel inflammation score (**B**) from LCWE-injected mice, or LCWE-injected mice that received either the platelet-depleting anti-CD42b antibody or IgG control at 1 week after LCWE injection (*n* = 7 to 8 mice/group). Scale bars: 200 μm. (**C**–**E**) Representative pictures of the abdominal aorta area (**C**), maximal abdominal aorta diameter (**D**), and abdominal aorta area measurements (**E**) from LCWE-injected mice, or LCWE-injected mice that received either the platelet-depleting anti-CD42b or IgG control at 1 week after LCWE injection (*n* = 7 to 8 mice/group). (**F**) Levels of IL-1B in the serum of PBS- or LCWE-injected mice that received either IgG isotype control or the platelet-depleting anti-CD42b antibody on day 1 or day 7 after LCWE injection (*n* = 4 to 7 mice/group). (**G**) Representative H&E staining and immunostaining staining of CD41 (red) in serial heart sections of WT mice injected with PBS, LCWE, or LCWE-injected mice treated with the platelet-depleting anti-CD42b. (**H**) CD41^+^ cell counts in the coronary artery of WT mice injected with PBS, LCWE, or LCWE-injected mice treated with the platelet-depleting anti-CD42b. Scale bars: 200 μm (H&E) and 100 μm (immunofluorescence). Each symbol represents 1 mouse. Results presented as mean ± SEM. *P < 0.05; **P < 0.01; ***P < 0.001; ****P < 0.0001 obtained by 1-way ANOVA with Tukey’s multiple-comparison test (**B** and **D**–**G**).

To determine whether increased platelet counts promote the development of LCWE-induced KD cardiovascular lesions, we investigated the effects of platelet depletion in LCWE-injected mice. Thrombocytopenia can be induced in mice by intravenous injection of anti-CD42b (anti-GP1ba) antibody (19, 22). WT mice were injected with either anti-CD42b or an IgG isotype control 6 hours before LCWE injection and again on day 3 after LCWE injection (Supplemental Figure 1A; supplemental material available online with this article; https://doi.org/10.1172/jci.insight.169855DS1). Treatment of LCWE-injected mice with anti-CD42b resulted in a significant decrease in frequencies and absolute numbers of blood platelets compared with either untreated mice or mice that received the IgG isotype control (Supplemental Figure 1, B and C). Remarkably, platelet depletion with anti-CD42b significantly reduced the severity of the cardiovascular inflammation and lesions (Figure 3, A and B). The development of LCWE-induced abdominal aorta aneurysms, measured by maximal abdominal aorta diameter and abdominal aorta area, was also decreased in anti-CD42b–treated platelet-depleted mice (Figure 3, C–E).

IL-1B signaling is required for LCWE-induced KD vasculitis, and blocking the NLRP3/IL-1 pathway in mice, either genetically by targeting the *Nlrp3*, *Il1b,* or *Il1* receptor genes, or pharmacologically using anakinra, IL-1 neutralizing antibodies, or NLRP3 small-molecule inhibitors, reduces the development of LCWE-induced cardiovascular lesions (7, 45–47). Monocytes and macrophages are the main sources of IL-1 during LCWE-induced KD vasculitis (7, 46, 47). Since platelets bind monocytes to form MPAs, boost their NLRP3 activation, and license monocytes and macrophages to produce higher levels of IL-1B (19), we quantified serum levels of IL-1B in LCWE-injected mice treated or not with anti-CD42b. As expected, LCWE injection increased the levels of serum IL-1B on days 1 and 7 after injection, but this was significantly reduced in anti-CD42b–treated thrombocytopenic mice (Figure 3F). Immunofluorescent staining of heart tissue sections with the classical platelet marker CD41 demonstrated elevated platelet numbers in proximity to the inflamed CA in LCWE-injected mice, which was normalized in anti-CD42b–treated mice (Figure 3, G and H). These results demonstrate that platelets promote the development of LCWE-induced cardiovascular lesions, potentially in an IL-1B–dependent mechanism.

### Deletion of c-Mpl inhibits LCWE-induced cardiovascular inflammation.

**Figure 4:**
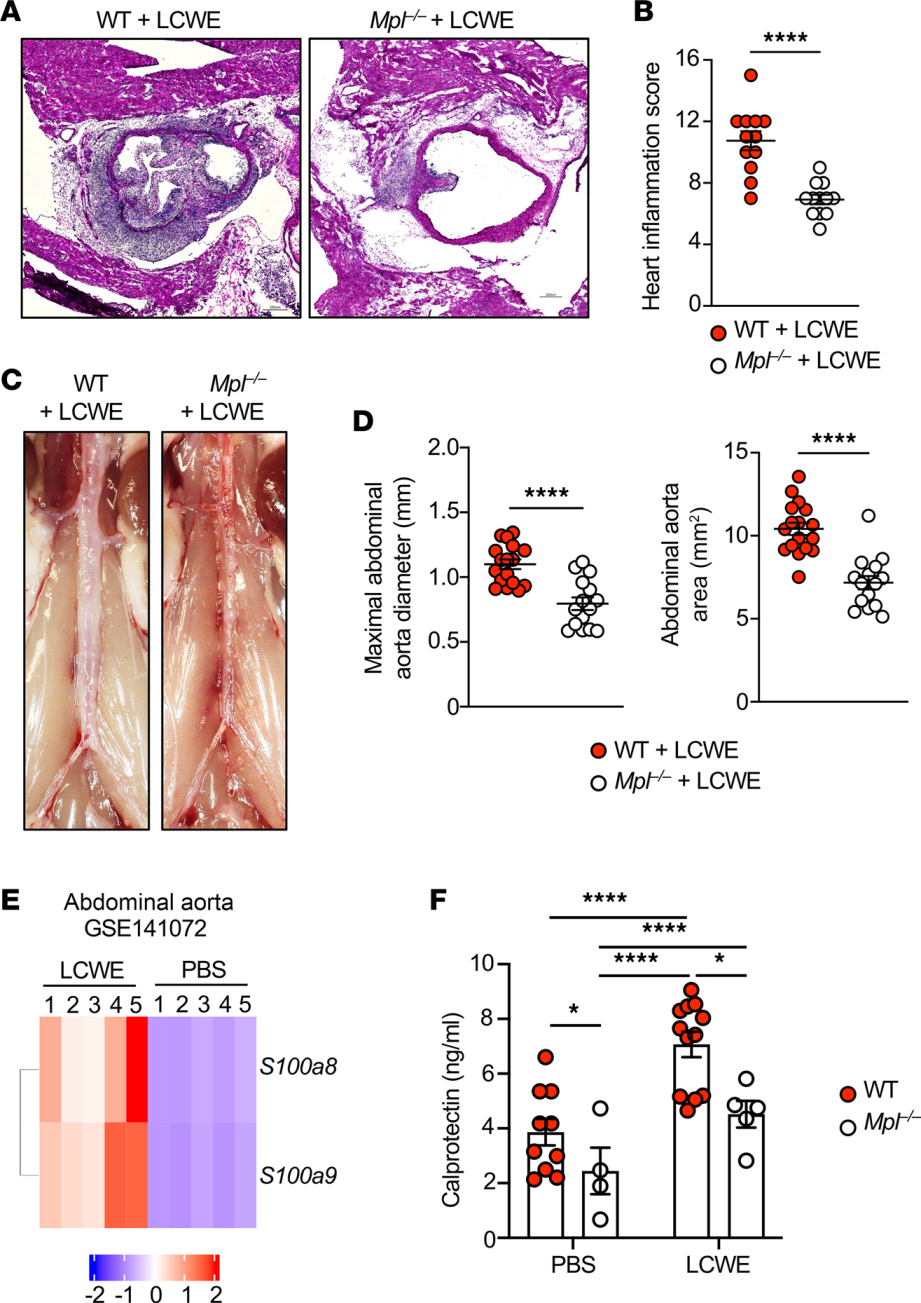
Decreased development of LCWE-induced cardiovascular lesions in thrombocytopenic *Mpl^–/–^* mice. (**A** and **B**) Representative H&E-stained heart tissue sections (**A**) and heart inflammation score (**B**) of LCWE-injected WT and *Mpl^–/–^* mice 1 week after LCWE injection (*n* = 12/group). Scale bars: 200 μm. (**C** and **D**) Representative pictures of the abdominal aorta area (**C**) and maximal abdominal aorta diameter and abdominal aorta area measurements (**D**) from WT and *Mpl^–/–^* mice (*n* = 15–17/group) at 1 week after LCWE injection. (**E**) Heatmap illustrating the expression of *S100a8* and *S100a9* (at least 1.5 FC with an adjusted *P* value < 0.05) in abdominal aorta tissues of PBS- and LCWE-injected mice (*n* = 5/groups; GSE141072). Blue–red color gradient: Low to high expression relative to the mean of each column. Each row represents 1 mouse of the defined groups. (**F**) Calprotectin levels in the serum of PBS- and LCWE-injected WT and *Mpl^–/–^* mice, at 1 week after injection (*n* = 4 to 12/group). Each symbol represents 1 mouse. Results presented as mean ± SEM pooled from 2–3 independent experiments. *P < 0.05; ****P < 0.0001 obtained by unpaired 2-tailed Student’s *t* test with Welch’s correction (**B**), unpaired 2-tailed Student’s *t* test (**D**), or 2-way ANOVA with Tukey’s multiple-comparison test (**F**).

We found that TPO, which stimulates thrombocytosis, was elevated in LCWE-injected mice (Figure 2E). TPO signals through the c-Mpl receptor, which is expressed by megakaryocyte progenitor cells, megakaryocytes, and platelets (56, 59). To further investigate the impact of platelets on LCWE-induced KD vasculitis in vivo, we used *Mpl^–/–^* mice, which are severely thrombocytopenic (60, 61). As previously reported, *Mpl^–/–^* mice developed normally, but when compared with WT mice, showed a significant decrease in blood CD61^+^ cells, a marker predominantly expressed by platelets and megakaryocytes (Supplemental Figure 2). Compared with WT mice, *Mpl^–/–^* mice showed reduced heart vessel inflammation following LCWE injection (Figure 4, A and B) and were relatively protected from the development of abdominal aorta dilations and aneurysms (Figure 4, C and D). Circulating levels of the alarmin calprotectin (S100A8/S100A9), which are usually increased in immune-mediated inflammatory diseases, are elevated in LCWE-injected mice and patients with KD (62). Indeed, analysis of publicly available gene expression data (GSE141072; ref. 63) showed that LCWE-injected mice had increased expression of *S100a8* and *S100a9* in their abdominal aorta compared with PBS-injected mice (Figure 4E). Since platelets might be a source of calprotectin (64, 65), we next assessed serum levels of calprotectin in WT and *Mpl^–/–^* mice injected with either PBS or LCWE. Compared with WT mice, thrombocytopenic *Mpl^–/–^* mice showed decreased levels of circulating calprotectin both at baseline and after LCWE injection (Figure 4F). These data indicate that the TPO-mediated increase in platelet count contributes to the development of cardiovascular lesions and that platelets may contribute to calprotectin production in the LCWE-induced murine model of KD vasculitis.

### MPAs promote increased IL-1B production during LCWE-induced KD vasculitis.

**Figure 5:**
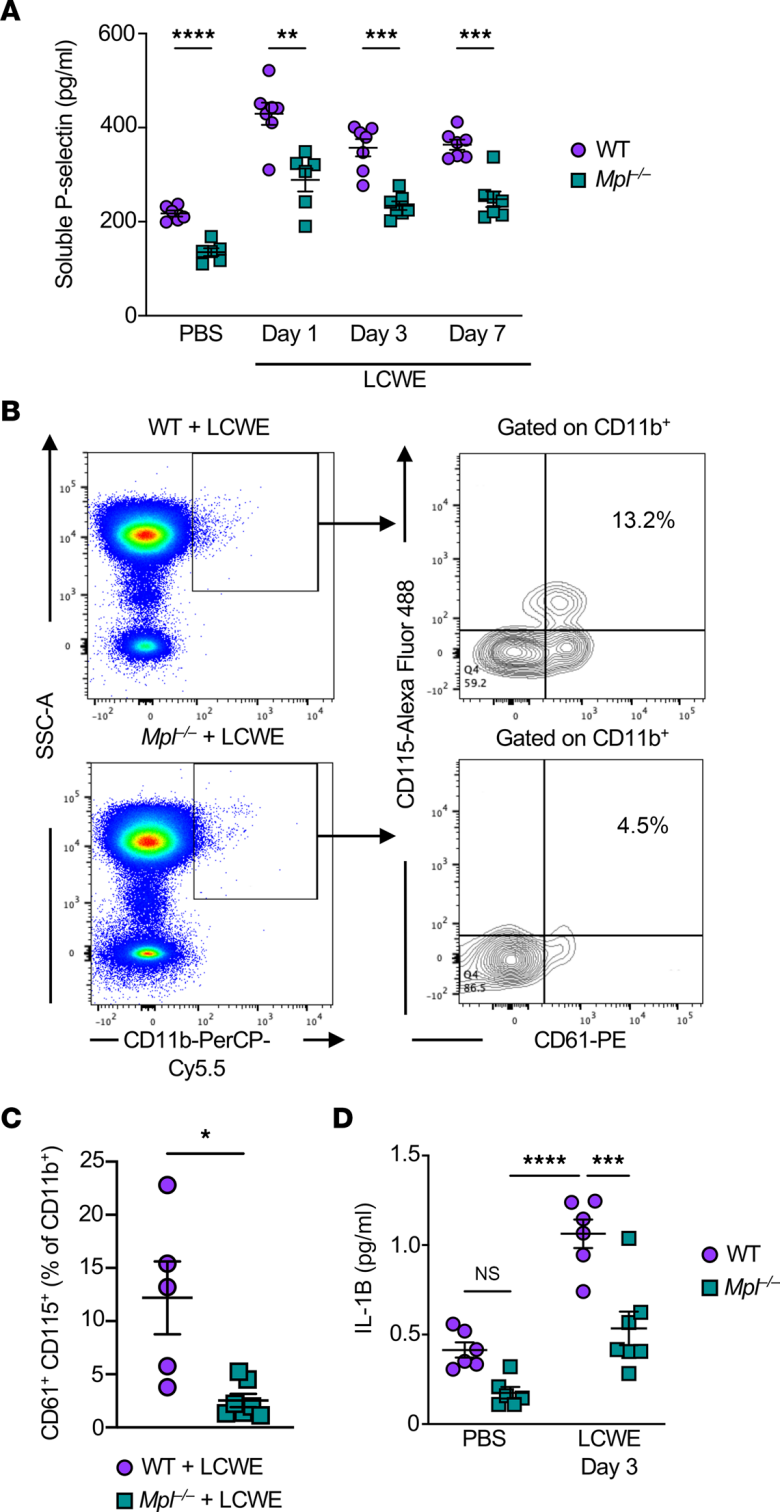
Increased frequencies of circulating monocyte-platelet aggregates (MPAs) during LCWE-induced KD vasculitis. (**A**) Serum levels of soluble P-selectin in PBS- or LCWE-injected WT and *Mpl^–/–^* mice (*n* = 6–7/group). (**B** and **C**) Representative flow cytometry plots (**B**) and frequencies (**C**) of CD61^+^CD11b^+^CD115^+^ MPAs in whole blood of WT and *Mpl^–/–^* mice 3 days after LCWE injection (*n* = 5–7/group). (**D**) Serum IL-1B concentration in PBS-and LCWE-injected WT and *Mpl^–/–^* mice at baseline and 3 days after injection (*n* = 6–7/group). Each symbol represents 1 mouse. Results presented as mean ± SEM. *P < 0.05; **P < 0.01; ***P < 0.001; ****P < 0.0001 obtained by 2-way ANOVA with Bonferroni’s multiple-comparison test (**A** and **D**) or 2-tailed Mann-Whitney test (**C**).

Since the level of soluble P-selectin, which is released by activated platelets, is elevated in patients with KD during the subacute phase of the disease (66, 67), we next measured the circulating levels of soluble P-selectin in LCWE-injected WT and *Mpl^–/–^* mice (Figure 5A). *Mpl* deficiency resulted in significantly reduced levels of soluble P-selectin at baseline and throughout the course of LCWE-induced vasculitis (Figure 5A). Notably, platelet clumps were visible on peripheral blood smears of LCWE-injected WT mice, starting 24 hours after injection (Supplemental Figure 3). Platelet-leukocyte aggregates, particularly those containing monocytes and neutrophils, are considered markers of inflammation in several disorders, including cardiovascular diseases (23, 68). Platelet-neutrophil aggregates have been reported in KD patients and shown to increase in frequency in patients with CA aneurysms (43). In addition, patients with KD also exhibit increased frequencies of MPAs (44). We defined MPAs in flow cytometry as CD11b^+^CD61^+^CD115^+^ and observed that the frequencies of MPAs were decreased in LCWE-injected *Mpl^–/–^* mice compared with WT mice (Figure 5, B and C). Since activated platelets have the capacity to boost inflammasome activation and IL-1B production by monocytes and neutrophils (19), we next measured serum levels of IL-1B in PBS- and LCWE-injected WT mice and thrombocytopenic *Mpl^–/–^* mice. While LCWE injection resulted in increased levels of circulating IL-1B in both WT and *Mpl^–/–^* mice, the magnitude of this elevation was significantly lower in *Mpl^–/–^* mice (Figure 5D). We acknowledge that even though we did not isolate platelets, it is possible that the method of blood collection for platelet count may potentially activate platelets and MPA formation. However, the same method was used and performed for all the experimental groups. Overall, these results support the notion that the increased platelet number promotes MPA formation and drives further IL-1B release in LCWE-induced KD vasculitis, which may exacerbate the development of cardiovascular lesions.

## Discussion

While platelets are known to be involved in the development and progression of many inflammatory and vascular diseases, their contribution to the development of the cardiovascular lesions of KD vasculitis has been unclear. The LCWE-induced murine model of KD vasculitis phenocopies the key immunopathologic features of KD, including coronary arteritis and luminal myofibroblast proliferation (32). This mouse model of KD vasculitis has helped establish the critical role of IL-1B in this disease, as blocking the IL-1 pathway either with anakinra or using small-molecule inhibitors of NLRP3, or using mice deficient in *Il1a*, *Il1b*, *Nlrp3*, or *Casp1*, is the most efficient way to decrease the severity of LCWE-induced KD vasculitis, and is more effective than targeting other proinflammatory mediators (7, 45–47). Using this model, we reveal increased platelet counts in the second week of LCWE-induced KD development, which correlates with the severity of the cardiovascular lesions. Furthermore, platelet depletion, either by an anti-platelet antibody or using thrombocytopenic *Mpl^–/–^* mice, results in decreased severity of LCWE-induced KD cardiovascular lesions. We also observed increased frequencies of MPAs and higher circulating levels of IL-1B during LCWE-induced KD vasculitis, which were reduced in thrombocytopenic mice. These data support the hypothesis that platelets may contribute to the development and severity of LCWE-induced cardiovascular lesions via an IL-1B–driven mechanism. We acknowledge that one limitation of our study is that we only measured platelet counts and did not investigate their function.

Previous studies have reported enhanced spontaneous platelet aggregation and elevated circulating levels of platelet activation markers during the acute phase of KD (37, 67, 69). Our analysis of publicly available transcriptomics data sets indicates that the expression of genes known to be either platelet specific or strongly associated with platelet activity increases in children with KD compared with HCs and decreases during the convalescent phase of the disease after IVIG therapy. In addition, KD patients with CA aneurysms exhibit higher platelet counts and increased circulating levels of the platelet activation marker β-thromboglobulin, hinting at the potential participation of platelets in the development of cardiovascular lesions (35, 42). However, the role of platelets in the development, progression, and severity of cardiovascular lesions during KD is not well understood, and the specific mechanisms by which platelets may contribute to KD pathology remain undetermined. KD is associated with systemic inflammation and endothelial dysfunction (1). Activated platelets release mediators, such as matrix metalloproteinases, growth factors, lipid mediators, cytokines, microvesicles, reactive oxygen species, galectins, TGF-β, and PF4, which may also regulate vascular remodeling by promoting the narrowing of the vessel wall and destruction of the CA (16, 17). Upon vascular injury or endothelial dysfunction, activated platelets adhere to the injured endothelium, and these platelet-released mediators may, therefore, influence vascular function and contribute to the remodeling and stenosis of the CA also observed in patients with KD. Levels of circulating endothelial cells, which detach from vessel walls and indicate vascular injury, are elevated in patients with KD compared with HCs, and this increase is even more pronounced in patients with KD who develop CA aneurysms (70, 71). We hypothesize that this vascular injury may lead to platelet activation, which initiates a vicious cycle by amplifying activation of the NLRP3 inflammasome and IL-1B production by monocytes and macrophages, and worsening vessel damage (19, 72). Indeed, we report a positive correlation between high platelet counts and the severity of LCWE-induced cardiovascular lesions and inflammation and reduced lesion formation in the setting of platelet depletion.

Our study suggests that inhibiting the formation of MPAs may have promising therapeutic potential for KD vasculitis, since MPA formation promotes the release of proinflammatory cytokines by monocytes. Hypothetically, MPA formation could be inhibited by targeting the binding of P-selectin on platelets and PSGL1 on monocytes. Indeed, it has been shown that in vitro, targeting of P-selectin or PSGL1 strongly decreases MPA formation (29, 73). Similarly, MPA formation can also be lowered in vitro by targeting the platelet receptor P2Y12 (73, 74). Currently, high-dose IVIG together with aspirin is the mainstay of therapy in acute KD (1, 2), but the role and the optimal dose of aspirin during the acute stage of the disease remain controversial. Furthermore, aspirin is inefficient in blocking the formation of platelet-leukocyte aggregates and MPAs, and does not regulate P-selectin levels (23, 75). In cases of severe or complex CA aneurysms with thrombotic complications, other antiplatelet drugs, such as clopidogrel, are recommended by the American Heart Association (1). Since MPAs participate in both thrombosis and inflammation, and their frequencies are increased in patients with KD (44), further studies aiming to determine the potential beneficial effect of pharmacologically targeting MPAs and their IL-1B production during KD are warranted.

Notably, however, platelets can also exert their proinflammatory effects on human macrophages and monocytes via a soluble mediator, in a mechanism that does not involve cell-to-cell contact (19, 29). Activated platelets release α-granule–derived cytokines, chemokines, and growth factors, and in vitro, platelet releasate increases the expression of inflammatory transcripts by human macrophages (73, 76). A recent study further supports the crucial role of platelet cytokine-driven transcription factors in licensing the inflammatory immune response of monocytes (29), as disassembling MPAs using a specific monoclonal antibody against human P-selectin does not affect the capacity of platelets to boost monocyte cytokine responses (29).

Circulating levels of the proinflammatory alarmin calprotectin (S100A8/A9 or MRP8/MRP14) are elevated in patients with KD during the early acute phase of the disease and decrease after IVIG treatment (77, 78). Furthermore, persistence of high levels of calprotectin after IVIG treatment correlates with the development of CA aneurysms (78), and IVIG-resistant KD patients have increased transcript abundance for *S100A8* and *S100A9* in whole blood when compared with IVIG-responsive KD patients (79). Purified monocytes from patients with KD before IVIG treatment exhibit increased mRNA expression of *S100A8* and *S100A9* (78), indicative of their capacity to produce calprotectin. However, calprotectin can also be released by other cell types, including activated neutrophils and platelets, and elevated calprotectin levels are associated with platelet activation (64, 72). Calprotectin concentrations are positively associated with both platelet aggregation and MPA formation in patients with coronary artery diseases (80, 81). In addition, in peripheral artery diseases, activated platelets are enriched in MRP14, which increases their P-selectin expression and the formation of MPAs, and promotes the monocyte inflammatory profile by increasing *IL1B*, *CCL2*, and *TNFA* mRNA levels (82). We previously reported that LCWE-injected mice have increased circulating levels of calprotectin (62), and here, we report decreased calprotectin levels in *Mpl^–/–^* mice. However, whether platelets are the main source of calprotectin or enhance its release from myeloid cells or other cellular sources during KD remains to be determined. Thus, calprotectin may represent another potential therapeutic target to modulate cardiovascular lesion formation in KD.

Taken together, our data indicate that platelets may participate in the inflammatory immune response during KD pathogenesis, including ongoing vascular inflammation and remodeling after the initial acute stage, and in the development and progression of cardiovascular lesions by increasing MPA formation and the release of IL-1B. Our results demonstrate the immune-effector role of platelets in KD and indicate that strategies targeting MPA formation might be beneficial for patients with KD in addition to blocking the IL-1B pathway. Future studies should be directed at better understanding the role of platelet-leukocyte aggregates in KD vasculitis, their potential use as a prognostic tool, and the therapeutic consequences of inhibiting their formation. These observations emphasize the role of IL-1B in the overall pathogenesis of KD vasculitis, providing additional mechanistic insight into elevated IL-1B production during KD and highlighting the need for phase III clinical trials with anti–IL-1 therapeutic agents, such as anakinra.

## Methods

### Animals.

WT C57BL/6J and *Mpl^–/–^* (C57BL/6J-*Mpl^hlb219^*/J) mice were purchased from the Jackson Laboratory. Experimental knockout animals were obtained from homozygous breeding, and age- and sex-matched WT mice from our internal colony at Cedars-Sinai were used as controls. Only male mice were used in this study, as LCWE injection induces stronger and more consistent cardiovascular lesions in male than female mice (46, 63). Mice were maintained under specific pathogen–free conditions and used according to the guidelines of the institutional animal care and use committee (IACUC) of Cedars Sinai Medical Center.

### LCWE-induced murine model of KD vasculitis.

*Lactobacillus casei* (ATCC 11578) cell wall extract (LCWE) was prepared as previously described (45). Five-week-old male mice were injected intraperitoneally (i.p.) with 500 μg of LCWE or an equal volume of PBS and euthanized 1 to 2 weeks after injection, as indicated in the figure legends. On the day of euthanasia, blood was collected via retro-orbital bleeding and centrifuged to obtain serum. Mice were then perfused with PBS and heparin through the heart left ventricle, and heart tissues were harvested and embedded in Tissue-Tek Optimum Cutting Temperature (O.C.T.) compound (Sakura Finetek, catalog 4583). Abdominal aortas were dissected, and photographed before embedding in Tissue-Tek O.C.T. The maximal abdominal aorta diameter was determined by measuring 5 different areas separated by 2 mm of the abdominal aorta infra-renal portion (below the left renal artery) with ImageJ (NIH). The infrarenal abdominal aorta area was also measured in ImageJ. Serial cryosections (7 μm) of heart tissues were stained with hematoxylin and eosin (H&E; MilliporeSigma, catalog MHS32). Heart tissue histopathological examination and assessment of the severity of cardiovascular lesions (CAs, aortic root vasculitis, and myocarditis) were performed on H&E-stained tissue sections by an expert pathologist blinded to the experimental groups, as previously described (45). Briefly, acute inflammation, chronic inflammation, and connective tissue proliferation were each assessed using the following scoring system: 0 = no inflammation, 1 = rare inflammatory cells, 2 = scattered inflammatory cells, 3 = diffuse infiltrate of inflammatory cells, and 4 = dense clusters of inflammatory cells. Fibrosis was determined using the following scoring system: 0 = no medial fibrosis, 1 = medial fibrosis involving less than 10% of the CA circumference, 2 = medial fibrosis involving 11% to 50% of the CA circumference, 3 = medial fibrosis involving 51% to 75% of the CA circumference, and 4 = medial fibrosis involving more than 75% of the CA circumference. All 4 scores were combined to generate a severity score called “Heart inflammation score,” as previously published (45).

### In vivo platelet depletion.

In vivo platelet depletion was performed using an anti-CD42b (anti-GP1ba) antibody (Emfret Analytics, catalog R300) or an isotype control IgG (rat IgG; Emfret Analytics, catalog C301). Antibodies were injected intravenously (i.v.) at a dose of 3 mg/kg body weight starting 6 hours prior to LCWE injection. Three days later, the administration of anti-CD42b or control IgG was repeated i.v. at a dosage of 4 mg/kg.

### Absolute quantification of platelet count.

Blood was obtained from mouse tail vein and collected in BD Microtainer blood collection tubes (BD Biosciences, catalog 365974). Platelet quantification was performed on 50 μL of mouse blood by the Department of Comparative Medicine at Cedars-Sinai Medical Center using a Drew Scientific Hemavet analyzer (GMI Inc.). When indicated, platelet quantification was performed by flow cytometry. Collected blood samples were stained with anti-CD61–PE (clone 2C9.G2; BioLegend, catalog 104307) or an IgG isotype control (clone HTK888; BioLegend, catalog 400907). Cell numbers were calculated by flow cytometry with CountBright Counting Beads (Thermo Fischer Scientific, catalog C36950) according to the manufacturer’s instructions. Stained samples were analyzed on an SA3800 Spectral Analyzer (Sony Biotechnology) and the data were processed using FlowJo (Tree Star Inc.).

### ELISAs.

Levels of TPO (R&D Systems, catalog MTP00), IL-6 (BD Biosciences, catalog 555240), S100A8/S100A9 heterodimer (R&D Systems, catalog DY8596-05), and P-selectin (R&D Systems, catalog DY737) were measured in the serum of PBS and LCWE-injected mice by ELISA according to the manufacturer’s protocol. IL-1B was quantified in serum using the V-PLEX Mouse IL-1B Assay (Meso Scale Diagnostics, catalog K152QPD-1) per the manufacturer’s instructions using the MSD QuickPlex SQ120 instrumentation and Workbench 4.0 Software (Meso Scale Diagnostics).

### Immunofluorescence.

Frozen heart tissues were collected from mice injected with either PBS, LCWE, or LCWE plus anti-CD42b. Serial cryosections (7 μm) of heart tissues were generated, fixed in acetone, and stained with anti-CD41–PE (clone MWReg30; BioLegend, catalog 133905) or a PE IgG isotype control (clone RTK2071; BioLegend, catalog 400907) antibodies to detect platelets in heart tissue sections. Images were obtained using a Biorevo BZ-9000 (Keyence) fluorescence microscope. The quantification of CD41-positive cells per mm^2^ in the tissues surrounding coronary arteries was done with ImageJ (NIH).

### Flow cytometric detection of MPAs.

Whole blood was collected from PBS- or LCWE-injected mice and stained with anti-CD61–PE (clone 2C9.G2; BioLegend, catalog 104307), anti-CD11b–PerCP-Cy5.5 (clone M1/70; Tonbo Biosciences, catalog 65-0112-U100), and anti-CD115–Alexa Fluor 488 (clone AFS98; eBioscience, catalog 53-1152-82). The following antibodies were used as isotype controls: hamster anti-IgG–PE (clone HTK888; BioLegend, catalog 400907), rat anti-IgG2b–PerCP-Cy5.5 (clone Ltf-2; Tonbo Biosciences, catalog 65-4031-U100), and rat anti-IgG2a–Alexa Fluor 488 (clone eBR2a; eBioscience, catalog 53-4321-80). Stained cells were analyzed on a FACSAria III (BD Biosciences) and the data were processed using FlowJo (Tree Star Inc.).

### Whole-blood smear.

Twenty-four hours after PBS or LCWE injection, a small drop of blood was placed on a glass slide and spread. The smear was allowed to dry and fixed in methanol followed by staining in Wright-Giemsa stain solutions Camco Quik Stain II (Thomas Scientific, catalog C872R02) according to the manufacturer’s instructions. Images were obtained with the Biorevo BZ-9000 (Keyence) fluorescence microscope.

### Analysis of gene expression data sets.

The publicly available gene expression data sets GSE68004 (52), GSE73461 (53), and GSE63881 (54) were obtained from the NCBI GEO (https://www.ncbi.nlm.nih.gov/geo/). Transcriptomic data analysis was run by GEO2R software as part of the GEO database, and summary statistics were generated with the limma topTable function. The following list of genes was selected as a “platelet gene signature,” as previously published (19): *C6orf25*, *CA2*, *CABP5*, *CXCL1*, *CXCL5*, *F13A1*, *F2RL1*, *GNG11*, *GP1BA*, *GP1BB*, *GP9*, *GRAP2*, *HIST1H2BK*, *HIST1H3H*, *ITGA2B*, *ITGB3*, *MYL9*, *NRGN*, *PDGFA*, *PDLIM1*, *PDZK1IP1*, *PF4*, *PF4V1*, *PLA2G7*, *PPBP*, *PRKAR2B*, *PTCRA*, *PTGS1*, *RSG18*, *SDPR*, *SELP*, *SMOX*, *SPARC*, *TBXA2R*, *TGFB1I1*, *THBS1*, *TMSB4X*, *TUBB1*, *VEGFA*, and *ZNF185*. This list of platelet signature genes was selected from direct comparisons of publicly available transcriptomes of purified human and mouse platelets (51, 83, 84) and reflects genes that are exclusively or abundantly expressed by platelets. Expression of the platelet gene signature was first assessed in whole blood of patients with KD and HCs (*n* = 37 HCs and *n=76* acute KD patients for GSE68004, ref. 52; *n* = 55 HCs and *n* = 77 acute KD patients for GSE73461, ref. 53). Differentially expressed genes (DEGs) (Benjamini-Hochberg–adjusted *P* < 0.05 and FC > 1.5) from the platelet gene signature were identified in these 2 data sets. DEGs in both data sets (*n* = 19; *CXCL1*, *F13A1*, *F2RL1*, *GNG11*, *GP9*, *HIST1H2BK*, *HIST1H3H*, *ITGA2B*, *ITGB3*, *MYL9*, *NRGN*, *PPBP*, *PTCRA*, *PTGS1*, *SDPR*, *SMOX*, *SPARC*, *TUBB1*, and *ZNF185*) were then selected, and their expression subsequently analyzed in another data set generated from whole blood of patients with acute KD (*n* = 146) and IVIG-treated convalescent KD patients (*n* = 145) based on a Benjamini-Hochberg–adjusted *P* value of less than 0.05 (GSE63881) (54). The expression of *S100a8* and *S100a9* transcripts was determined in a murine RNA-Seq data set generated from abdominal aortas of PBS-injected (*n* = 5) and LCWE-injected (*n* = 5) mice (GSE141072) (63). Normalization and analysis of gene expression data were performed in R using edgeR and limma-voom, as previously published (63). *S100a8* and *S100a9* genes were considered DEGs, with an adjusted *P* value of less than 0.05 and FC of 1.5 or greater. Heatmaps showing the expression relative to the mean of selected genes were generated in R with the “ComplexHeatmap” package.

### Statistics.

Statistical analysis was performed using Prism software (GraphPad). Data normality was determined using the Shapiro-Wilk normality test, with α = 0.05. For 2-group comparisons, unpaired, 2-tailed Student’s *t* test was used for normally distributed data, and 2-tailed Mann-Whitney test was used for non–normally distributed data or for groups with *n* less than 7. For more than 2 groups, 1-way ANOVA with Tukey’s post hoc analysis was used for normally distributed data. For multiple-comparison testing, significance was evaluated by 2-way ANOVA with Bonferroni’s post hoc test. Correlations were calculated with the Pearson’s correlation coefficient. Results are reported as mean ± SEM, where each point represents 1 sample. A *P* value of less than 0.05 was considered statistically significant. All schematics and the graphical abstract were done with Biorender.

### Study approval.

All animal studies in this paper were approved by the IACUC of Cedars Sinai Medical Center and were performed in accordance with the NIH *Guide for the Care and Use of Laboratory Animals* (National Academies Press, 2011).

### Data availability.

Data supporting the findings of this study are available from the corresponding author upon reasonable request. The publicly available gene expression data sets GSE68004 (52), GSE73461 (53), and GSE63881 (54) were obtained from NCBI GEO (https://www.ncbi.nlm.nih.gov/geo/). The murine RNA-Seq data set generated from the abdominal aortas of PBS- and LCWE-injected mice (GSE141072) (63) was downloaded from NCBI GEO (https://www.ncbi.nlm.nih.gov/geo/). The platelet gene signature was selected from direct comparisons of publicly available transcriptomes of purified human and mouse platelets (51, 83, 84) and reflects genes that are exclusively or abundantly expressed by platelets. The remaining data are available within the article or supplemental information.

## Author contributions

BK, NN, M Abe, MNR, and M Arditi conceptualized the study. BK, YL, NN, M Abe, D Martinon, MEL, D Moreira, SC, and RAP performed experiments. MNR and M Arditi supervised experiments. Data analysis was performed by BK, YL, NN, M Abe, and MCF. Data discussion was contributed by BK, NN, M Abe, SC, RAP, BSF, MNR, and M Arditi. Manuscript writing was contributed by BK, YL, BSF, MNR, and M. Arditi. The order of equally contributing authors was decided by contribution, seniority and funding of the study.

## Supplementary Material

Supplemental data

## Version 1 06/06/2023

In-Press Preview

## Version 2 07/24/2023

Electronic publication

## Version 3 08/18/2023

The labels for Figure 2E and 2F were swapped. The labels are fixed in this version

## References

[1] McCrindle BW (2017). Diagnosis, treatment, and long-term management of Kawasaki disease: a scientific statement for health professionals from the American Heart Association. Circulation.

[2] Soni PR (2020). A comprehensive update on Kawasaki disease vasculitis and myocarditis. Curr Rheumatol Rep.

[3] Rowley AH (2018). Is Kawasaki disease an infectious disorder?. Int J Rheum Dis.

[4] Skochko SM (2018). Kawasaki disease outcomes and response to therapy in a multiethnic community: a 10-year experience. J Pediatr.

[5] Maury CP (1988). Circulating interleukin-1 beta in patients with Kawasaki disease. N Engl J Med.

[6] Alphonse MP (2016). Inositol-triphosphate 3-kinase C mediates inflammasome activation and treatment response in Kawasaki disease. J Immunol.

[7] Porritt RA (2021). NLRP3 inflammasome mediates immune-stromal interactions in vasculitis. Circ Res.

[8] Dinarello CA (2012). Treating inflammation by blocking interleukin-1 in a broad spectrum of diseases. Nat Rev Drug Discov.

[9] Weber A (2010). Interleukin-1 (IL-1) pathway. Sci Signal.

[10] Blonz G (2020). Severe late-onset Kawasaki disease successfully treated with anakinra. J Clin Rheumatol.

[11] Guillaume MP (2018). Usefulness and safety of anakinra in refractory Kawasaki disease complicated by coronary artery aneurysm. Cardiol Young.

[12] Kone-Paut I (2018). The use of interleukin 1 receptor antagonist (anakinra) in Kawasaki disease: a retrospective cases series. Autoimmun Rev.

[13] Koné-Paut I (2021). Phase II open label study of anakinra in intravenous immunoglobulin-resistant Kawasaki disease. Arthritis Rheumatol.

[14] Coppinger JA (2004). Characterization of the proteins released from activated platelets leads to localization of novel platelet proteins in human atherosclerotic lesions. Blood.

[15] Ruggeri ZM, Mendolicchio GL (2007). Adhesion mechanisms in platelet function. Circ Res.

[16] Semple JW (2011). Platelets and the immune continuum. Nat Rev Immunol.

[17] Aggarwal A (2023). Platelets at the vessel wall in non-thrombotic disease. Circ Res.

[18] Koupenova M (2022). Platelet and megakaryocyte roles in innate and adaptive immunity. Circ Res.

[19] Rolfes V (2020). Platelets fuel the inflammasome activation of innate immune cells. Cell Rep.

[20] Morrell CN (2019). The platelet napoleon complex-small cells, but big immune regulatory functions. Annu Rev Immunol.

[21] Schrottmaier WC (2020). Platelet-leukocyte interplay during vascular disease. Atherosclerosis.

[22] Barrett TJ (2019). Platelet regulation of myeloid suppressor of cytokine signaling 3 accelerates atherosclerosis. Sci Transl Med.

[23] Allen N (2019). Circulating monocyte-platelet aggregates are a robust marker of platelet activity in cardiovascular disease. Atherosclerosis.

[24] Freedman JE, Loscalzo J (2002). Platelet-monocyte aggregates: bridging thrombosis and inflammation. Circulation.

[25] Rinder HM (1991). Dynamics of leukocyte-platelet adhesion in whole blood. Blood.

[26] Frenette PS (2000). P-Selectin glycoprotein ligand 1 (PSGL-1) is expressed on platelets and can mediate platelet-endothelial interactions in vivo. J Exp Med.

[27] Bournazos S (2008). Monocyte functional responsiveness after PSGL-1-mediated platelet adhesion is dependent on platelet activation status. Arterioscler Thromb Vasc Biol.

[28] Martins PAdC (2006). Platelet binding to monocytes increases the adhesive properties of monocytes by up-regulating the expression and functionality of β_1_ and β_2_ integrins. J Leukoc Biol.

[30] Hottz ED (2020). Platelet activation and platelet-monocyte aggregate formation trigger tissue factor expression in patients with severe COVID-19. Blood.

[31] Ridker PM (2017). Antiinflammatory therapy with canakinumab for atherosclerotic disease. N Engl J Med.

[32] Noval Rivas M, Arditi M (2020). Kawasaki disease: pathophysiology and insights from mouse models. Nat Rev Rheumatol.

[33] (1986). Kawasaki disease and the plight of the platelet. Am J Dis Child.

[34] Arora K (2020). Platelets in Kawasaki disease: is this only a numbers game or something beyond?. Genes Dis.

[35] Levin M (1985). Platelet immune complex interaction in pathogenesis of Kawasaki disease and childhood polyarteritis. Br Med J (Clin Res Ed).

[36] Wei M (2015). A multicenter study of intravenous immunoglobulin non-response in Kawasaki disease. Pediatr Cardiol.

[37] Taki M (2003). Spontaneous platelet aggregation in Kawasaki disease using the particle counting method. Pediatr Int.

[38] Jin J (2019). Platelet-derived microparticles: a new index of monitoring platelet activation and inflammation in Kawasaki disease. Indian J Pediatr.

[39] Ueno K (2010). Platelet vascular endothelial growth factor is a useful predictor for prognosis in Kawasaki syndrome. Br J Haematol.

[40] Zhang Y (2020). Reduced platelet miR-223 induction in Kawasaki disease leads to severe coronary artery pathology through a miR-223/PDGFRβ vascular smooth muscle cell axis. Circ Res.

[41] Straface E (2010). Oxidative stress and defective platelet apoptosis in naïve patients with Kawasaki disease. Biochem Biophys Res Commun.

[42] Burns JC (1984). Coagulopathy and platelet activation in Kawasaki syndrome: identification of patients at high risk for development of coronary artery aneurysms. J Pediatr.

[43] Ueno K (2015). Circulating platelet-neutrophil aggregates play a significant role in Kawasaki disease. Circ J.

[44] Vignesh P (2021). Monocyte platelet aggregates in children with Kawasaki disease- a preliminary study from a tertiary care centre in North-West India. Pediatr Rheumatol Online J.

[45] Lee Y (2012). Interleukin-1β is crucial for the induction of coronary artery inflammation in a mouse model of Kawasaki disease. Circulation.

[46] Wakita D (2016). Role of interleukin-1 signaling in a mouse model of Kawasaki disease-associated abdominal aortic aneurysm. Arterioscler Thromb Vasc Biol.

[47] Lee Y (2015). IL-1 signaling is critically required in stromal cells in Kawasaki disease vasculitis mouse model: role of both IL-1α and IL-1β. Arterioscler Thromb Vasc Biol.

[48] Takahashi K (2005). Neutrophilic involvement in the damage to coronary arteries in acute stage of Kawasaki disease. Pediatr Int.

[49] Takahashi K (2011). Pathogenesis of Kawasaki disease. Clin Exp Immunol.

[50] Simon LM (2014). Human platelet microRNA-mRNA networks associated with age and gender revealed by integrated plateletomics. Blood.

[51] Rowley JW (2011). Genome-wide RNA-seq analysis of human and mouse platelet transcriptomes. Blood.

[52] Jaggi P (2018). Whole blood transcriptional profiles as a prognostic tool in complete and incomplete Kawasaki disease. PLoS One.

[53] Wright VJ (2018). Diagnosis of Kawasaki disease using a minimal whole-blood gene expression signature. JAMA Pediatr.

[54] Hoang LT (2014). Global gene expression profiling identifies new therapeutic targets in acute Kawasaki disease. Genome Med.

[55] Noval Rivas M (2017). CD8^+^ T cells contribute to the development of coronary arteritis in the Lactobacillus casei cell wall extract-induced murine model of Kawasaki disease. Arthritis Rheumatol.

[56] Kaushansky K (2005). The molecular mechanisms that control thrombopoiesis. J Clin Invest.

[57] Kaser A (2001). Interleukin-6 stimulates thrombopoiesis through thrombopoietin: role in inflammatory thrombocytosis. Blood.

[58] Ishiguro A (1998). Elevation of serum thrombopoietin precedes thrombocytosis in Kawasaki disease. Thromb Haemost.

[59] Li J (1999). Interaction of thrombopoietin with the platelet c-mpl receptor in plasma: binding, internalization, stability and pharmacokinetics. Br J Haematol.

[60] Gurney AL (1994). Thrombocytopenia in c-Mpl-deficient mice. Science.

[61] Alexander WS (1996). Deficiencies in progenitor cells of multiple hematopoietic lineages and defective megakaryocytopoiesis in mice lacking the thrombopoietic receptor c-Mpl. Blood.

[62] Noval Rivas M (2019). Intestinal permeability and IgA provoke immune vasculitis linked to cardiovascular inflammation. Immunity.

[63] Porritt RA (2020). Interleukin-1 beta-mediated sex differences in Kawasaki disease vasculitis development and response to treatment. Arterioscler Thromb Vasc Biol.

[64] Wang Y (2014). Platelet-derived S100 family member myeloid-related protein-14 regulates thrombosis. J Clin Invest.

[65] Barrett TJ (2021). Platelets amplify endotheliopathy in COVID-19. Sci Adv.

[66] Takeshita S (1997). Circulating soluble selectins in Kawasaki disease. Clin Exp Immunol.

[67] Furui J (2002). Soluble forms of the selectin family in children with Kawasaki disease: prediction for coronary artery lesions. Acta Paediatr.

[68] Gerrits AJ (2016). Whole blood analysis of leukocyte-platelet aggregates. Curr Protoc Cytom.

[69] Yahata T (2014). Platelet activation dynamics evaluated using platelet-derived microparticles in Kawasaki disease. Circ J.

[70] Shah V (2015). Cardiovascular status after Kawasaki disease in the UK. Heart.

[71] Nakatani K (2003). Circulating endothelial cells in Kawasaki disease. Clin Exp Immunol.

[72] Scherlinger M The role of platelets in immune-mediated inflammatory diseases. Nat Rev Immunol.

[73] Rolling CC (2023). P2Y12 inhibition suppresses proinflammatory platelet-monocyte interactions. Thromb Haemost.

[74] Liverani E (2012). Prednisolone exerts exquisite inhibitory properties on platelet functions. Biochem Pharmacol.

[75] Klinkhardt U (2003). Clopidogrel but not aspirin reduces P-selectin expression and formation of platelet-leukocyte aggregates in patients with atherosclerotic vascular disease. Clin Pharmacol Ther.

[76] Kral JB (2016). Platelet interaction with innate immune cells. Transfus Med Hemother.

[77] Hirono K (2006). Expression of myeloid-related protein-8 and -14 in patients with acute Kawasaki disease. J Am Coll Cardiol.

[78] Abe J (2005). Gene expression profiling of the effect of high-dose intravenous Ig in patients with Kawasaki disease. J Immunol.

[79] Fury W (2010). Transcript abundance patterns in Kawasaki disease patients with intravenous immunoglobulin resistance. Hum Immunol.

[80] Larsen SB (2015). Calprotectin and platelet aggregation in patients with stable coronary artery disease. PLoS One.

[81] Song NP (2020). Plasma calprotectin was associated with platelet activation and no-reflow phenomenon in acute coronary syndrome. BMC Cardiovasc Disord.

[82] Dann R (2018). Platelet-derived MRP-14 induces monocyte activation in patients with symptomatic peripheral artery disease. J Am Coll Cardiol.

[83] Eicher JD (2016). Characterization of the platelet transcriptome by RNA sequencing in patients with acute myocardial infarction. Platelets.

[84] McRedmond JP (2004). Integration of proteomics and genomics in platelets: a profile of platelet proteins and platelet-specific genes. Mol Cell Proteomics.

