# Supplementary material for: Platelets exacerbate cardiovascular inflammation in a murine model of Kawasaki disease vasculitis
Source: JCI Insight. 2023 Jul 24;8(14):e169855. doi: 10.1172/jci.insight.169855 (PMC10443810; doi:10.1172/jci.insight.169855)
Supplement: Supplemental data [file jciinsight-8-169855-s262.pdf]

Supplementary Figure 1

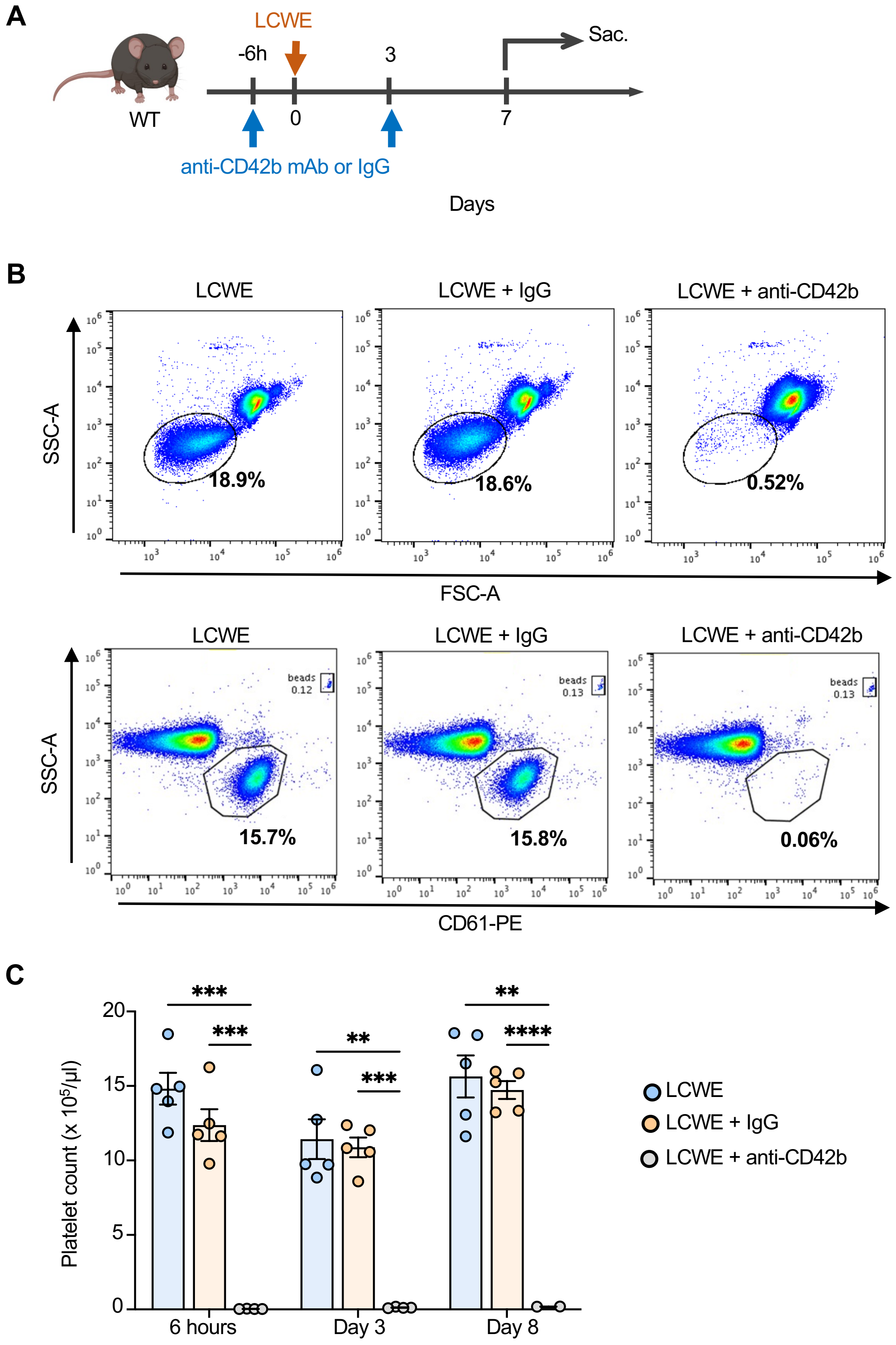

Supplementary Figure 1. Treatment with anti-CD42b significantly reduces platelet counts in LCWE-injected mice. (**A**) Schematic representation of the experimental design. WT mice were left either untreated or received anti-CD42b or an IgG isotype 6 hours prior LCWE-injection and again 3 days after the LCWE injection. One week post-LCWE injection, heart tissues and abdominal aortas were collected. (**B**) Representative flow cytometry plots showing the frequencies of platelets in the blood of mice injected with LCWE and treated with either the platelet-depleteing antibody anti-CD42b or IgG control at day 7 post-LCWE injection (**C**) Absolute number of platelets in the blood of mice injected with LCWE and treated with either the platelet-depleting antibody anti-CD42b or IgG control. Each symbol represents one mouse. Results presented as mean ± s.e.m. **p<0.01, ***p<0.001, ****p<0.0001 obtained by 2-way ANOVA with Bonferonni's multiple comparisons test.

Supplementary Figure 2

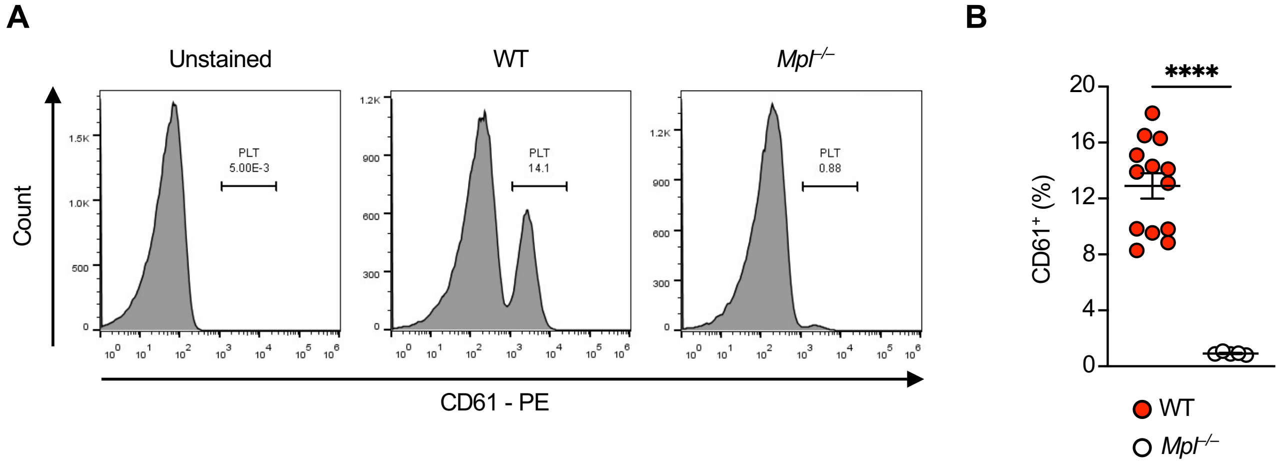

Supplementary Figure 2. Severe thrombocytopenia in *Mpl*–/– mice. (**A, B**) Representative flow plots (A) and CD61+ cell quantification (B) in the blood of WT and *Mpl*–/– mice (n=5 to 13 mice /group). Each symbol represents one mouse. Results presented as mean ± s.e.m. ****p<0.0001 obtained by Unpaired t-test with Welch's correction.

Supplementary Figure 3

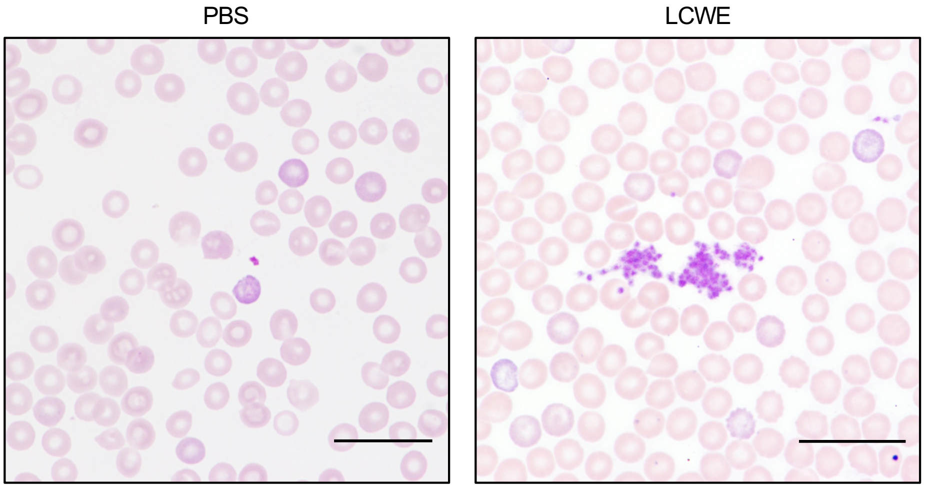

Supplementary Figure 3. Platelet aggregates during LCWE-induced KD vasculitis. Representative pictures of blood smears from PBS- or LCWE-injected WT mice at 24 hours after injection. Scale bars: 20μm.
